# Supplementary material for: Conditional chemoconnectomics (cCCTomics) as a strategy for efficient and conditional targeting of chemical transmission
Source: eLife. 2024 Apr 30;12:RP91927. doi: 10.7554/eLife.91927 (PMC11060718; doi:10.7554/eLife.91927)
Supplement: Figure 3—source data 3. [file elife-91927-fig3-data3.zip › Figure 3 source data3 part 2.zip!/Figure 3-Source data 3-part2/1052-13-CRISPResso_Report_ejedac2.zip!/CRISPResso_on_1052_13/4d.Position_dependent_average_indel_size.pdf]

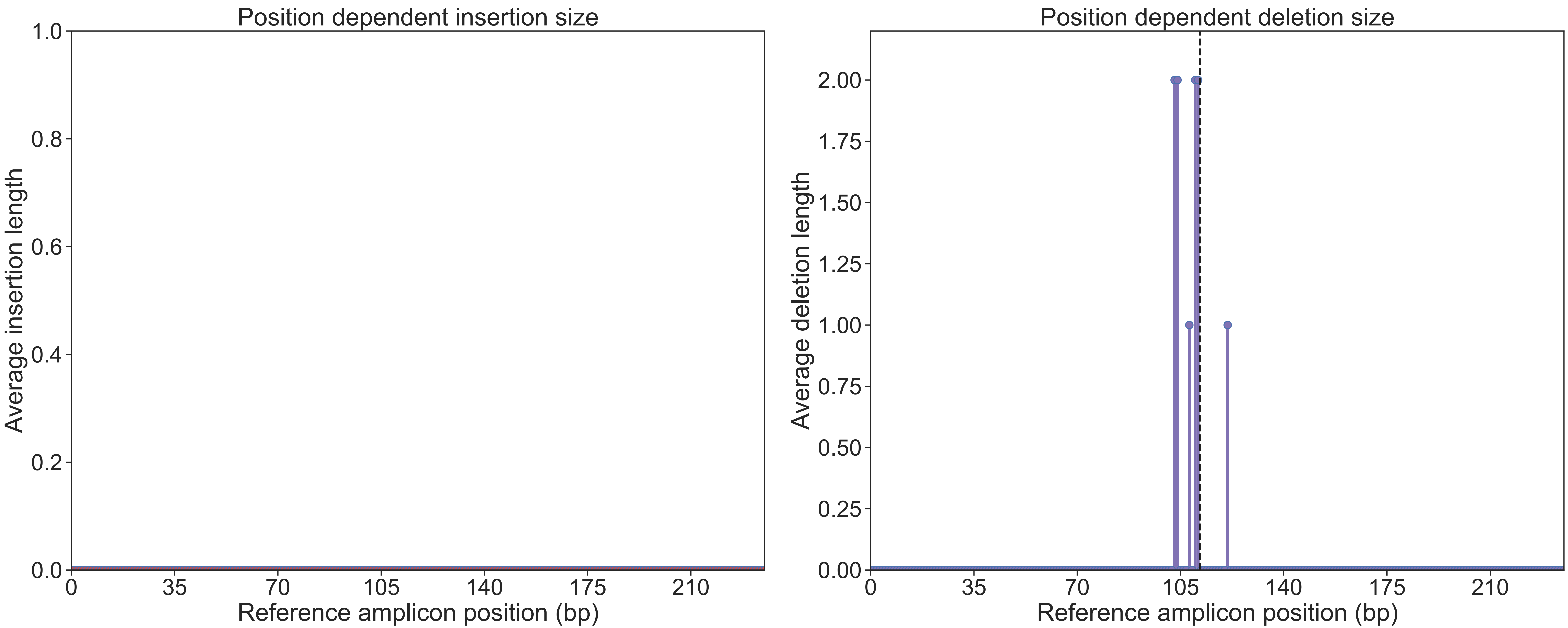

Position dependent insertion size
Average insertion length
1.0
0.8
0.6
0.4
0.2
0.0
0
35
70
105
140
175
210
Reference amplicon position (bp)
Position dependent deletion size
Average deletion length
2.00
1.75
1.50
1.25
1.00
0.75
0.50
0.25
0.00
0
35
70
105
140
175
210
Reference amplicon position (bp)
